# Detecting climate adaptation with mobile network data in Bangladesh: anomalies in communication, mobility and consumption patterns during cyclone Mahasen

**DOI:** 10.1007/s10584-016-1753-7

**Published:** 2016-08-01

**Authors:** Xin Lu, David J. Wrathall, Pål Roe Sundsøy, Md. Nadiruzzaman, Erik Wetter, Asif Iqbal, Taimur Qureshi, Andrew J. Tatem, Geoffrey S. Canright, Kenth Engø-Monsen, Linus Bengtsson

**Affiliations:** 1 Department of Public Health Sciences, Karolinska Institutet, Stockholm, Sweden; 2 Flowminder Foundation, Stockholm, Sweden; 3 College of Information System and Management, National University of Defense Technology, Changsha, China; 4 College of Earth, Ocean and Atmospheric Sciences, Oregon State University, Corvallis, OR USA; 5 Telenor Research, Oslo, Norway; 6 Department of Geography, University of Exeter, Exeter, UK; 7 International Centre for Climate Change and Development, Dhaka, Bangladesh; 8 Stockholm School of Economics, Stockholm, Sweden; 9 WorldPop, Department of Geography and Environment, University of Southampton, Southampton, UK

**Keywords:** Climate change adaptation, Migration, Resilience, Mobile network data, Anomaly detection, Disaster risk

## Abstract

Large-scale data from digital infrastructure, like mobile phone networks, provides rich information on the behavior of millions of people in areas affected by climate stress. Using anonymized data on mobility and calling behavior from 5.1 million Grameenphone users in Barisal Division and Chittagong District, Bangladesh, we investigate the effect of Cyclone Mahasen, which struck Barisal and Chittagong in May 2013. We characterize spatiotemporal patterns and anomalies in calling frequency, mobile recharges, and population movements before, during and after the cyclone. While it was originally anticipated that the analysis might detect mass evacuations and displacement from coastal areas in the weeks following the storm, no evidence was found to suggest any permanent changes in population distributions. We detect anomalous patterns of mobility both around the time of early warning messages and the storm’s landfall, showing where and when mobility occurred as well as its characteristics. We find that anomalous patterns of mobility and calling frequency correlate with rainfall intensity (*r* = .75, *p* < 0.05) and use calling frequency to construct a spatiotemporal distribution of cyclone impact as the storm moves across the affected region. Likewise, from mobile recharge purchases we show the spatiotemporal patterns in people’s preparation for the storm in vulnerable areas. In addition to demonstrating how anomaly detection can be useful for modeling human adaptation to climate extremes, we also identify several promising avenues for future improvement of disaster planning and response activities.

**Electronic supplementary material:**

The online version of this article (doi:10.1007/s10584-016-1753-7) contains supplementary material, which is available to authorized users.

## Introduction

The increasingly robust evidence base in climate sciences relies on the measurement of normal trends and analysis of deviations (Bindoff et al. 2013). Techniques for detecting anomalies have produced key findings on changing atmospheric and surface temperature (Jones et al. 1999; Mann et al. 1998), oceanic circulation (Hurrell 1995; Thompson and Wallace 1998), arctic temperatures and ice cover (Serreze et al. 2000; Stroeve et al. 2007; Vinje 2001), intensity of tropical rainfall and cyclones (Knutson et al. 2010; Trenberth 2011); and seasonal variability and extremes (Seneviratne et al. 2012). Anomaly detection principles have also shown how earth’s ecosystems (Lucht et al. 2002; Stenseth et al. 2002) and biota including agriculture (Lenoir et al. 2008) have responded to climate change. However, human behavior in response to disasters also deviates from normal behavioral patterns. In this paper, we aim to use anomaly detection to investigate behavioral responses in a human population exposed to an extreme weather event.

Vulnerable people in low- and middle-income countries respond to weather extremes associated with climate change, such as tropical cyclones and flooding, with a variety of behaviors that appear anomalous against a baseline (here termed “adaptations”) such as moving animals to safety, harvesting crops early, reinforcing and repairing flood embankments, and changing household spending behaviors. In more extreme cases, short-term adaptive responses include evacuation and displacement. Weather extremes can, in the long-term, undermine livelihoods, push people into poverty, and elicit an extraordinary adaptive response in these circumstances: permanent migration (Black et al. 2011; Brouwer et al. 2007), a subject of rich academic debate (summarized in (Black et al. 2013) featured centrally in 5th Assessment of the IPCC (Adger et al. 2014; Olsson et al. 2014). Unfortunately, our ability to detect anomalous human behaviors is not on par with our large-scale measurements of biophysical systems at relevant temporal and spatial scales.

Climate science has seen rapid progress in the measurement, and prediction of changes and extremes in biophysical systems in high resolution across geographic and temporal scales. To understand the impacts of climate change on human society it is imperative to measure anomalous behavioral responses as they coincide with hazards at the common spatiotemporal scales in which they occur (Palmer and Smith 2014). This is especially crucial where people are dependent on stable environmental conditions for livelihoods, and where both climate change and the burden of adaptation threaten human security and development (Adger et al. 2014; Field et al. 2014). Methodologies that focus on large-scale spatial indicators of both human behavioral and environmental change, and make use of temporally adjusted longitudinal data are required to establish baselines and link short-term responses and long-term outcomes (Palmer and Smith 2014).

As of the end of 2014, mobile networks served a total of 3.6 billion unique mobile subscribers, roughly half of the global population (GSMA Intelligence 2015). Mobile operator data are updated in close to real-time and have a vast geographic reach. The data generated from mobile operators enable measurement of some characteristics of social networks, migration, and patterns of household economic behavior at a previously unprecedented scale (Bagrow et al. 2011; Palmer and Smith 2014; Zolli 2012). Operator data has been used during relief operations after the Haiti 2010 earthquake (Bengtsson et al. 2011; Lu et al. 2012) and cholera outbreaks (Bengtsson et al. 2015) and the Nepal 2015 earthquake (Wilson et al. 2016), making them a very promising proxy indicator for measuring impacts of climate change, and weather extremes. In Rwanda, retrospective analyses of network activity was used to estimate the epicenter of an earthquake and to infer humanitarian needs in the weeks after the earthquake (Kapoor et al. 2010). Likewise, Blumenstock and colleagues identified unusual patterns of person-to-person transfers of airtime credits through social networks to identify a geographical pattern of earthquake impact (Blumenstock et al. 2011). Anomaly detection methods have previously been applied to mobile network data to identify unusual calling patterns after floods (Pastor-Escuredo et al. 2014), and in the interest of improving the normal operation of mobile networks (Karatepe and Zeydan 2014). They have been used for anomaly detection for detecting and classifying social disturbances, like conflict and violence in data-poor circumstances (Dobra et al. 2014; Young et al. 2014). One study showed the diffusion of anomalous calling patterns through intimate social networks in the wake of a terrorist bombing in Oslo (Sundsøy et al. 2012). Various studies have concluded that in the wake of disasters anomaly detection could reduce the cost, increase timeliness and improve the geographic focus of emergency response activities (Candia et al. 2008; Pawling et al. 2007).

The extreme South of coastal Bangladesh, with its low elevation and routine exposure to intense tropical cyclones, exemplifies an area with high climate pressure and is a fitting location to explore mobile network data before and after climate shocks. We searched for anomalous patterns of phone usage that could provide insight into adaptive preparations and responses (Martin et al. 2014; McGranahan et al. 2007; Penning-Rowsell et al. 2013), and examined how spatial and temporal patterns in large sets of operator data from the Grameenphone mobile network in Bangladesh around tropical cyclone Mahasen could inform impact assessment and adaptation in cyclone affected areas. We investigated three hypotheses. First, anomalous patterns of calling frequency represent the affected populations’ physical contact with the storm in the most affected areas during landfall. Second, as communication is an important tool during an environmental crisis, we hypothesized that anomalous mobile recharges represent behaviors of people preparing for impacts in the most vulnerable areas. Finally, we hypothesized that cyclones drive anomalous flows of users between towers, indicating evacuation, displacement and migration.

## Cyclone Mahasen

Cyclone Mahasen struck Bangladesh on 16 May 2013. Before landfall it moved northward along the Bay of Bengal. Forecasts estimated a landfall in the heavily populated Chittagong District, and the government’s Comprehensive Disaster Management Programme concentrated early warnings there. However, in the final hours of 15 May, the storm veered to the north, making landfall over the rural Barisal Division, at approximately 3:00 a.m. on 16 May (Fig. S1a). During the course of 16 May, Mahasen moved eastward along the coast into Chittagong, and northward into India, where rainfall and wind speed rapidly diminished (Gutro and Pierce 2013).

Mahasen was a relatively weak storm compared to earlier cyclones in Bangladesh, such as Aila and Sidr (REACH Initiative 2013). While it affected an estimated 1.3 million people (REACH Initiative 2013) and impacts on crops and homes were extensive, the death toll was relatively small. Seventeen perished in the storm, mostly from falling trees, and unlike previous storms, no fishermen were lost (Associated Press 2013). The minimal loss of life was regarded as a major victory for the Comprehensive Disaster Management Programme’s early warning system (UNDP 2013).

## Cyclone impacts and population-level adaptation

### Mobile phone data

We used a de-identified set of call detail records (CDRs) from 5.1 million Grameenphone users collected between 1 April and 30 June of 2013 in the Barisal Division and Chittagong District of Bangladesh. The dataset began six weeks before the landfall of Cyclone Mahasen (16 May 2013) and continued for six weeks after landfall (1 April to 30 June 2013) (Fig. S1). CDRs are compiled by network operators principally for the purposes of billing customers for their use of the network. De-identified data entries include information on the time of the call, the mobile phone tower used and the duration of call, and can thus be used to indicate the geographical position and movements of users. To limit potential biases resulting from subscriber churn, and new users entering the dataset due to impacts of the storm, we limited the study to SIM cards that had placed at least one call before the cyclone landfall (16 May); and also made at least one call in the last ten days of the data collection period (21–30 June).

Since Mahasen was a relatively weak cyclone, the performance of the Grameenphone network remained virtually undisturbed during and after landfall, guaranteeing continuous relay of CDR throughout the study period. An analysis of tower function anomalies appears in the *Supporting Information* (S2), along with a general discussion on the Grameenphone network, the dataset, and the representativeness of data for the general population (S1).

### Calling frequency and rainfall measurements

**Fig. 1:**
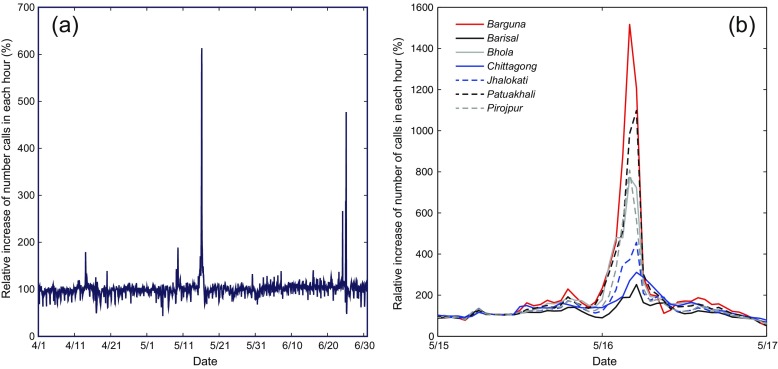
Change in call frequency. **a** For each hour, the number of calls is compared to the average number of calls made during that same hour across the whole period. Relative calling frequency spikes on 16 May as Mahasen makes landfall. **b** Calling frequency during cyclone landfall at the district level. For each of the seven districts, the change of calling frequency in the morning of 16 May is shown

During “normal” circumstances, which we defined as the average calls per hour for any given hour across the data set, regular daily and weekly cycles of calls were apparent (Fig. 1a). Users concentrated phone usage in the daytime hours, with a spike occurring toward late evening. A small shift in the temporal distribution of calls occurred on Fridays (the first day of the weekend) when calls began later in the day. Increases in calling frequency coincided with several events within the data set, most notably on 25 June, when a major religious festival, Shab-e-Barat was celebrated. Likewise smaller increases coincided with the Bengali New Year in early April and a series of protests in early May.

However in the early hours of Thursday 16 May 2013, as Mahasen made landfall across Barisal, we observed a dramatic increase in call frequency relative to “normal,” which we defined as calls per hour compared with the same hour on all Thursdays in the dataset (Fig. 1a). Deconstructing calling frequency among the more vulnerable coastal districts (Barguna, Bhola, Patuakhali, Pirojpur), we saw calling volumes increase by at least seven times the average level (Fig. 1b). In Barguna, calling frequency increased by a factor of 15. Throughout the evening and early hours, a spatiotemporal pattern emerged in peak calling frequency. In Barguna and Pirojpur, in the extreme south and west of Barisal, peaks occurred between 3:00 and 4:00 a.m., while in the northern districts of Jhalokhati and Barisal, the peak occurred between 4:00 and 5:00 a.m. This suggests that call frequency corresponded with the physical manifestations of the cyclone as it moved over Barisal Division from the South West. One alternative explanation is that people might call friends and relatives when a cyclone is approaching in order to communicate concerns for wellbeing, encourage evacuation plans, and coordinate preparations. However, between 00:00 and 6:00 a.m. at the time Mahasen was making landfall in Barisal, in the areas where the cyclone was predicted to make landfall (the Chittagong district), calling frequency was close to normal levels. These differences provide support for the hypothesis that calling frequency represented a behavioral response to sensory experience of the storm.

**Fig. 2:**
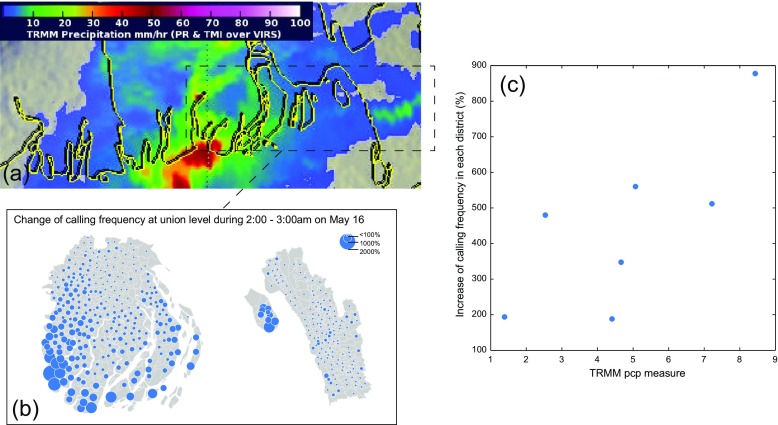
Call anomalies and rainfall. **a** Precipitation measurements from NASA’s Tropical Rainfall Measurement Mission captured at 3:32 a.m. show the distribution of rainfall in the study area, reprinted from Gutro and Pierce 2013, with permission from the authors. **b** The geographical distribution of call frequency at 3:00 a.m. on 16 May. **c** Rainfall is plotted with calling frequency at the district level. Correlation coefficient = 0.75, *p* = 0.05

To further investigate the relationship between calling frequency and physical manifestations of the storm, we conducted a spatiotemporal comparison of calling frequency with rainfall data from NASA’s Tropical Rainfall Measurement Mission (TRMM) satellite. The TRMM satellite passed over Bangladesh at 3:32 a.m., measuring rainfall during the cyclone’s landfall, reaching 67 mm per hour in some areas (Gutro and Pierce 2013). Locations of maximum rainfall were clearly correlated with locations of maximum increase in calling frequency (Fig. 2). Even areas with moderate rainfall, for example a narrow band of rainfall to the east of Chittagong (Fig. 2a) also exhibited an increase in calling frequency (Fig. 2b). This adds supporting evidence that clusters of high calling frequency represented contact with the cyclone’s most severe physical effects.

Past research has shown that rainfall data alone is often too low resolution and intermittent to make any inferences about cyclone damage (Auffhammer et al. 2013). Detailed spatiotemporal data on call frequency may improve inferences about the effect of weather extremes on vulnerable people, and is identified here as an area for future research.

### Recharge behaviors

**Fig. 3:**
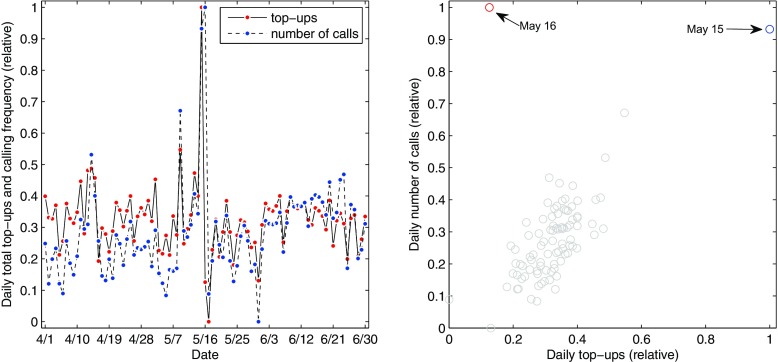
Comparison of daily recharges and calling frequency, both features are presented with min-max normalization. **a** During the cyclone, while calling frequency is high for both 15 May and 16 May, recharges are high only on 15 May and then drop to low levels on 16 May. **b** Excluding 16 May, there is a strong linear relationship between recharges and calling frequency (corr without 16 May = 0.798, *p* < 0.000, corr with 16 May = 0.576, *p* < 0.000), implying that recharges indicate users preparing for a potential disaster

Next, we investigated how mobile recharges or top-ups can complement call frequency to provide insight on how vulnerable people prepare for climate impacts. To accomplish this, we relied on a second data set, consisting of mobile recharge purchases from 892 retailers in Barisal and Chittagong Divisions during the original three-month timeline, 1 April to 30 June of 2013 (Fig. 3). Recharges are the amount of money that users credit to their SIM card to access the network. They allowed an investigation of the geographic distribution of changes in expenditures before and after the cyclone. In Bangladesh, mobile credits represent a small but significant proportion (~3 %) of the household budget (Lucini and Hatt 2014), and disasters increase demand for private communication (Samarajiva 2005). We hypothesized that spikes in recharges represented knowledge of the cyclone and preparations for its impacts.

In the second half of 15 May, as forecasts and early warnings were transmitted across radio and television, a large increase in recharges is evident, coinciding with a high volume of calls placed on the same day (Fig. 3). However even as calling frequency remained high on 16 May, recharges dropped below the predicted level, and continued at low levels during the following day. This suggests that users recharged their phones as part of their storm preparation and awareness of vulnerability, planning for the need to communicate with family and friends during and after the cyclone.

### Estimating evacuation, displacement and migration

**Fig. 4:**
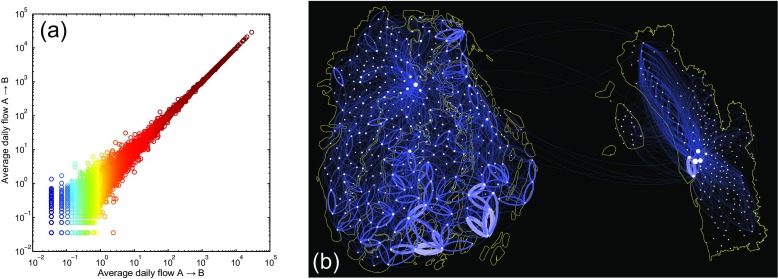
Symmetry in “normal” flow networks. **a** The average daily flow between each union in both directions between April 1, to April 28 (i.e., for four “normal” weeks prior to the storm) are shown. The correlation is extremely high (corr = 0.999, *p* < 0.000), indicating a high level of symmetry in “normal” mobility patterns, i.e. each day, roughly as many people leave an area as those who enter it. **b** This symmetry of “normal” flow can be represented geospatially. SIM cards are included only if they accessed more than one tower in a day. Links indicate areas where 10 or more movements were observed at distances greater than 10 km. Direction of flow is clockwise from the point of origin

**Fig. 5:**
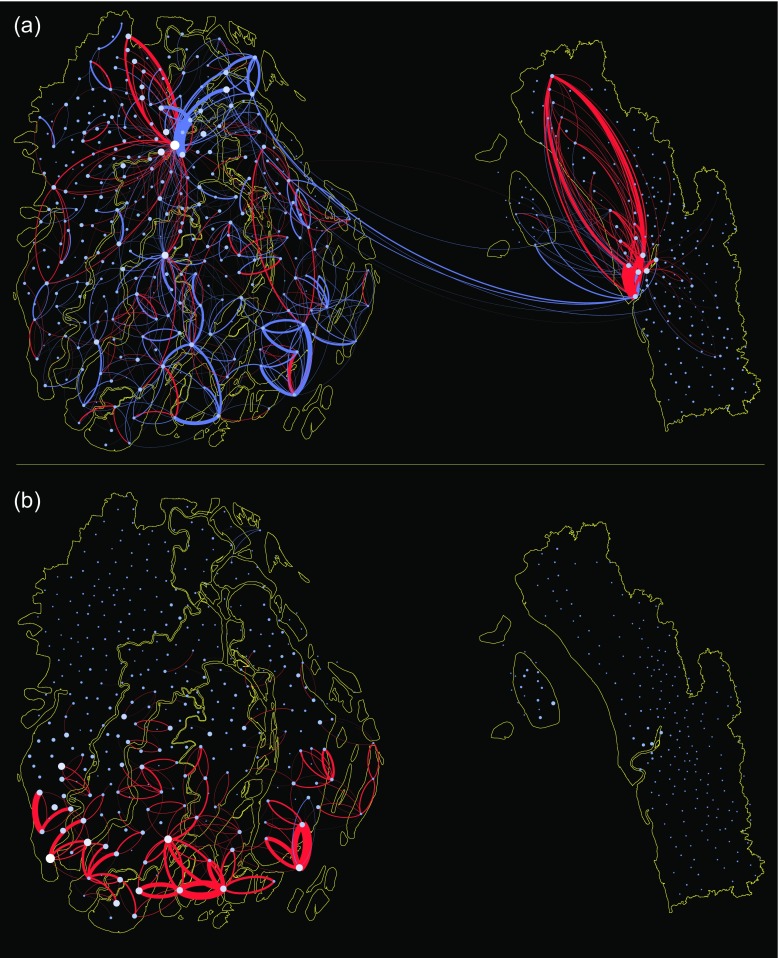
Evacuation and landfall flow networks. **a** The mobility network on 15 May (the day prior to landfall) is compared with 24 April (3 weeks before the storm during the same hourly period). Positive flows are shown in *red*, indicating increased flow on 15 May, while negative flows are shown in *blue*, indicating decreased flow on 15 May. Thickness of link represents relative volume of flow. To appear in the flow network, a user had to make at least two calls. Each SIM contributed only one movement (the first and last observed location). Links indicate areas where 10 or more movements were observed, at distances greater than 10 km. **b** The mobility network during landfall on 16 May, 00:00–6:00 a.m., is compared with 25 April (3 weeks prior during the same hourly period). Unusual mobility is observed in the affected area, where warnings were not concentrated

Usage patterns in the data also enabled us to analyse short-term features of evacuation, displacement and migration, which would be extremely hard to quantify using standard survey-based research but were readily apparent in CDRs. Using CDRs and tower locations to identify moving SIM cards, we created a series of mobility networks, which quantify the direction, volume and distance of flows between locations at specified time intervals before and after Cyclone Mahasen (Figs. 4 and 5). Note that the mobility networks during normal periods were almost perfectly symmetrical, meaning that the numbers of users entering an area are roughly equal to the number of users leaving an area (Fig. 4). In contrast, anomalies appeared as larger than normal flows in one or both directions (Fig. 5), and indicated spatiotemporally explicit patterns of movement, such as evacuation, displacement and permanent migration that took place at specific moments coinciding with the storm. Because asymmetrical flows might also represent, for example, the onset of migration season, a calendar festival or a popular protest, it is important to be cautious in assigning causation.

Prior to the storm, large changes in the flow network were notable in Chittagong City, as people evacuated in response to the forecasts that Mahasen would make landfall over Chittagong (Fig. 5a) Meanwhile, there were less than normal flows in Barisal at the same time, suggesting people were not evacuating to other areas in large numbers, but rather suspending regular trips.

In the early hours of May 16, during cyclone landfall, at the time when people should have been in shelters, above normal flows of SIMs were evident in the margin of sub-districts in Barisal nearest to the coast, indicating that people were moving about at night, during the storm (Fig. 5b). This suggests that people evacuated too late, and would have been in danger if the storm intensity had been greater. Mobility patterns in Barisal during landfall contrasted sharply with mobility in Chittagong during the same time, where patterns were virtually unchanged from normal flows for that day and hour. Although the exact explanation for these differences is unknown, officials in the Ministry of Disaster Planning and Response indicated that early warnings were not made in Barisal until too late because all forecasting indicated that the storm would make landfall in Chittagong (Nadiruzzaman 2013). Other possible explanations for delayed evacuation in rural areas were that men commonly stay behind to look after livestock and protect homes and assets from thieves.

In sum, the mobility patterns evident in mobile network data allow researchers to perform an audit of early warning program effectiveness on the basis of early and mid-storm population movements. In this case, the early warning system in Chittagong apparently accomplished the aim of motivating evacuation during appropriate times.

## Quantifying impacts and behavioral responses using anomaly detection techniques

To automatically detect human behavioral changes in our study, we used a sigma-model to evaluate the stability of the observed sequence of activities extracted from customers’ usage data in the mobile network. Specifically, for each time series of a quantified activity, E = {*e*_1_, *e*_2_, *e*_3_,  ... , *e*_*t*_},in which *e*_*i*_ ∈ R(1 ≤ *i* ≤ *t*) is the measure of the activity (e.g., number of calls at time *i*, and *t* the length of evaluated time window) we highlight time points I = {1 ≤ *i*_1_, *i*_2_,  ... , *i*_*M*_ ≤ *t*} in which each observation ${e}_{i_m}$ at time point *i*_*m*_ exceeds three standard deviations from E’s average during the time period.

**Fig. 6:**
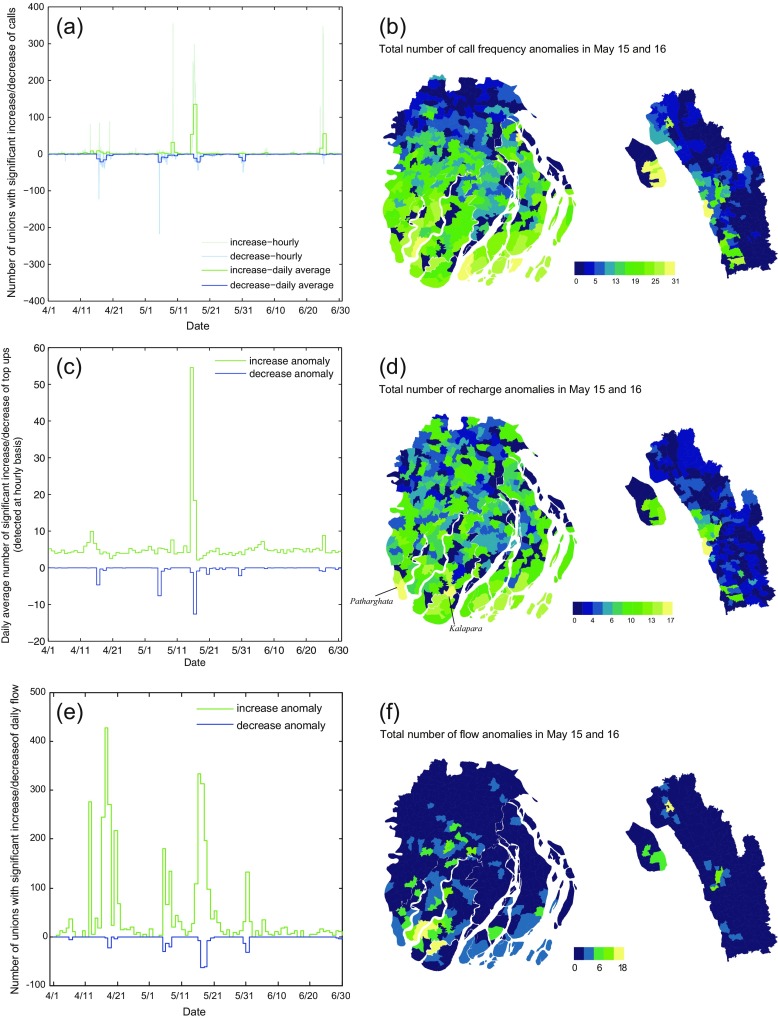
The temporal and spatial distribution of anomalies in calling frequency (**a**, **b**), recharge anomalies(**c**, **d**), and flow anomalies (**e**, **f**). The threshold for detection was set at three standard deviations from the mean of baseline. In the flow anomaly analysis, the restriction for including flows in the investigation was that they were positive for each day in the timeline

As the studied activity reached the predetermined thresholds, three standard deviations from the mean, it was flagged as anomalous. In this way, any unusual patterns of network usage could be identified, and further analysis would determine what these anomalies represented about cyclone impacts. When the number of anomalous cases is very large, the procedure may result in considerable false positives (type I error) (Candia et al. 2008). To avoid this limitation, we also calculated the total number of anomalies detected (see Fig. 6a).

### Anomaly detection for calling frequency

Unusual calling patterns provide a measure of behavioral response to storm severity. In the timeline, several clusters of calling frequency anomalies were observed (Fig. 6). The first occurred on 14 April, the Bengali New Year, followed by a drop the following day. A second cluster occurred on 9 May around an infamous series of national protests, dubbed “the Siege of Dhaka” in which several dozen people were killed across the nation in a series of violent protests. The next two clusters coincided with Mahasen, which made landfall on 15 and 16 May, and a cold front, which flooded the southern coast between 30 May and 1 June. Finally a large spike on 25 June coincided with Shab-e-Barat, an important religious festival, when people commonly call their relatives.

The most profound and longest lasting set of anomalies coincided with Mahasen. With few exceptions, calling frequency anomalies were concentrated along the vulnerable coastline, and in areas where the storm made landfall (Fig. 6b). These anomalies spatiotemporally coincided with cyclone landfall, and indicated when and where behavioral response to the physical cyclone were strongest.

Typically, post-cyclone damage assessments, which determine the form that disaster relief should take, rely on the reporting of damage by local officials. These rapid reports typically form the basis for selection of areas in which more detailed information on impacts and needs may be collected through household surveys. However systematic biases and delays can be introduced at various stages of the assessment process (Hallegatte and Przyluski 2010) due to limited capacity of responding agencies in many resource constrained settings. This analysis indicates how mobile network data could be used to overcome potential biases in the site selection portion of post-cyclone damage assessments by indicating when and where impacts have occurred.

### Anomaly detection relating to recharge behavior

Mobile recharge anomalies differ from other anomalous behaviors detected in the timeline in two ways: they are almost exclusively concentrated around the cyclone impact zone, and occured before the event, indicating foreknowledge of the coming cyclone and preparation for its impacts. As with calling frequency, recharge anomalies were concentrated along the vulnerable coastline, where the cyclone first made landfall (Fig. 6). But whereas calling frequency anomalies were detected widely across the region, mobile recharge anomalies were concentrated in Kalapara and Patharghata (Fig. 6d), areas noted as pockets of exceptional vulnerability within this already vulnerable landscape (Ahamed et al. 2012), where impacts of Cyclones Sidr (2007) and Aila (2009) were most severe. This suggests that anomalous preparation behaviors can reveal the areas where people perceive themselves to be vulnerable (Fig. 6d).

Alternative explanations include that these were areas where people had greater access to recharge vendors and spending money, however if this were the case, similar anomalies would have been observed in other urban centers of similar size. It is plausible that people undertook other anticipatory actions in areas where recharge anomalies were detected, but further study is required to link mobile recharges to overall disposition toward cyclone preparedness.

### Anomaly detection relating to population movements

Forms of human mobility that deviate from normal patterns can also be detected from mobile network data (Fig. 6). To identify anomalous flows, we investigated daily flow between each pair of locations (unions) during the whole period. If the daily flow between unions A and B exceeded three standard deviations from the mean for that weekday during the study period, a signal was generated for both unions A and B. Since a substantial number of location pairs normally have low flows, false alarms could result from small absolute increases. While anomalous decreases in daily flow were less likely to produce false alarms, there is potentially more noise in increases due to the small flows which normally pass between some locations. For simplicity, to decrease noise and to obtain a conservative measure of flow anomalies, we therefore focused on pairs of locations having non-zero flow during all days during the whole period.

As with call frequency anomalies, the flow anomalies detected in this analysis corresponded with the significant events in the time line: Bengali New Year (14 April), nation-wide protests (8 May), Cyclone Mahasen (16 May), and heavy rainstorms (30 May) (Fig. 6e). The largest cluster of anomalous flow increases coincided with the Bengali New Year, where very few anomalous flow decreases were simultaneously observed. Both anomalous flow increases and decreases were apparent between 15 and 19 May, before and after Mahasen struck, nevertheless the frequency of anomalous increases was eight times that of decreases. Note that the areas with most anomalous flow events coincided very well with the area in which rainfall intensity was the highest during cyclone landfall (Fig. 2).

## Conclusion

In this paper, we show how data from mobile networks provides insights into behavioral responses to Cyclone Mahasen and its impacts. We show that anomalous patterns of calling frequency are correlated with rainfall intensity at local scales, likely providing a defined spatiotemporal measure of users’ physical exposure to the storm. We show that mobile recharge purchases increase in vulnerable impact zones before landfall, representing preparations for potential environmental hazards. We also identify anomalous patterns of mobility during evacuation and storm landfall, indicating how people respond to storm forecasts and early warnings. The analysis is in agreement with the official joint needs assessment, which saw little evidence of mass displacement. We also show how, in future applications, anomalous flows of SIM cards between mobile towers can provide a much needed audit of the effectiveness of forecasting and early warnings systems, and indicate the new locations of displaced people. Rapid, cost-effective and accurate tools for assessing the effectiveness of early warning systems, and indicating the location of displaced people are currently in short supply.

Based on comparisons with rainfall measurements at landfall, and considering the substantial weakening of cyclones as they pass over land, calling frequency and population movement anomalies seemed to provide the best proxy indicators for cyclone impacts among those evaluated. Traditional methods for assessing cyclone impacts and human behavioral responses have well known limitations (Hallegatte and Przyluski 2010), and the anomaly detection technique applied to mobile network data presented here (building on work of Blumenstock et al. 2011; Candia et al. 2008; Dobra et al. 2014; Kapoor et al. 2010; Pawling et al. 2007; Sundsøy et al. 2012, and Young et al. 2014), may overcome some of these challenges, and demonstrates the potential value of mobile network data as a complement to current cyclone impact assessment tools. Specifically, the spatiotemporal distributions of anomalous usage activity could be used to improve the timeliness and cost-effectiveness of cyclone impact assessments. Data from mobile networks may be very useful as a tool to prioritize locations in which rapid needs assessments are performed after cyclone landfall, with the potential to drastically reduce the time to reach those most in need.

While the study provided a robust analysis of the behavior of Grameenphone subscribers, the primary limitations of the study involved the representativeness of data for the general population. However, the general features of behavior change that we found to be most useful, i.e. sharp increases in calling frequency and changes in mobility, may well result independently of mobile operator and are likely to reflect natural human responses to shocks. Likewise, the study concentrated on Mahasen, a relatively Cyclone, which despite maximum rainfall of 68 mm/h dissipated quickly. Findings cannot be generalized about larger, more energetic cyclones, where storm surges and flooding can cause greater destruction. Finally, other causes of increased calling frequency and mobility than those indicating a need for post-disaster assistance may exist after a disaster, and thus network data should, at our present level of understanding, be used as a complement to, not a replacement for, other information sources.

To overcome these limitations and to better understand the effects of multiple types of environmental disruption, future work should incorporate mobile network data covering longer time spans. Longitudinal household measures of storm impacts and improved environmental impact models can provide external validation of the spatiotemporal patterns of anomalous usage that are apparent in the mobile network data. Additionally, as we illustrate in the Supporting Information (S2), analysis of other aspects of network function, such as service interruptions, which do not convey information on human behavior, may still provide a proxy for spatiotemporal damage to infrastructure.

Detecting anomalous usage patterns from mobile network data is a promising avenue for researching human behavioral responses to impacts associated with climate change across large spatiotemporal scales. Data from mobile networks may become an important tool for prioritizing areas for rapid needs assessments following cyclones.

## Electronic Supplementary Material

ESM 1 (DOCX 1909 kb)
