# Supplementary material for: Detecting climate adaptation with mobile network data in Bangladesh: anomalies in communication, mobility and consumption patterns during cyclone Mahasen
Source: Clim Change. 2016 Aug 1;138(3):505–19. doi: 10.1007/s10584-016-1753-7 (PMC7175666; doi:10.1007/s10584-016-1753-7)
Supplement: Supplementary file 1 — (DOCX 1909 kb) [file 10584_2016_1753_MOESM1_ESM.docx]

**Supporting Information:**

**Detecting climate adaptation with mobile network data in Bangladesh: Anomalies in communication, mobility and consumption patterns during Cyclone Mahasen**

*Xin Lu ^a,b,c^, David J. Wrathall^d^, Pål Roe Sundsøy^e^, Md. Nadiruzzaman^f, g^,*

*Erik Wetter^b,h^, Asif Iqbal^e^, Taimur Qureshi^e^, Andrew Tatem^b,i^, Geoffrey S. Canright^e^,*

*Kenth Engø-Monsen^e^, Linus Bengtsson^a,b*^*

*^a^Department of Public Health Sciences, Karolinska Institutet, Stockholm, Sweden;
^b^Flowminder Foundation, Stockholm, Sweden;*

*^c^College of Information System and Management, National University of Defense Technology, Changsha, China;*

*^d^Oregon State University, College of Earth, Ocean and Atmospheric Sciences, Corvallis, Oregon, USA; ^e^Telenor Research, Oslo, Norway;*

*^f^Department of Geography, University of Exeter, Exeter, UK;*

*^g^International Centre for Climate Change and Development, Dhaka, Bangladesh;*

*^h^Stockholm School of Economics, Stockholm, Sweden;*

*^i^WorldPop, Department of Geography and Environment, University of Southampton, Southampton, UK.*

^*^Linus Bengtsson: [linus.bengtsson@flowminder.org](mailto:linus.bengtsson@flowminder.org)

S1. Dataset Characteristics

De-identified call detail records (CDRs) from 5.1 million Grameenphone users were collected between 1 April and 30 June of 2013 in the Barisal Division and Chittagong District of Bangladesh. The data begins six weeks before the landfall of Cyclone Mahasen (16 May 2013) and continues for six weeks after landfall (1 April to 30 June 2013) (Fig. S1a). CDRs are compiled by network operators principally for the purposes of billing customers for their use of the network. De-identified data entries include information on the time of the call, the mobile phone tower used and the duration of call, and can thus be used to indicate the geographical position and movements of users. To limit potential biases resulting from subscriber churn, and new users entering the dataset due to impacts of the storm, we limited the study to SIM cards that had placed at least one call before the cyclone landfall (16 May); and also made at least one call in the last ten days of the data collection period (21-30 June) .

A distribution of 986 towers across the Barisal Division and Chittagong District forms the basis for our spatiotemporal analysis of calling frequency, mobility and top-up behaviors. We assigned users a position within the network based on the tower through which his or her most recent call was routed (Fig. S1a). The coverage area of a mobile tower (BTS, base transceiver station) can range from a few hundred meters in a city, up to tens of kilometers in rural areas. The location of tower positions were further scrambled within 200 meters. The average and maximum distance of each tower and its nearest neighbor tower, were 2.1 km and 16.8 km, respectively.


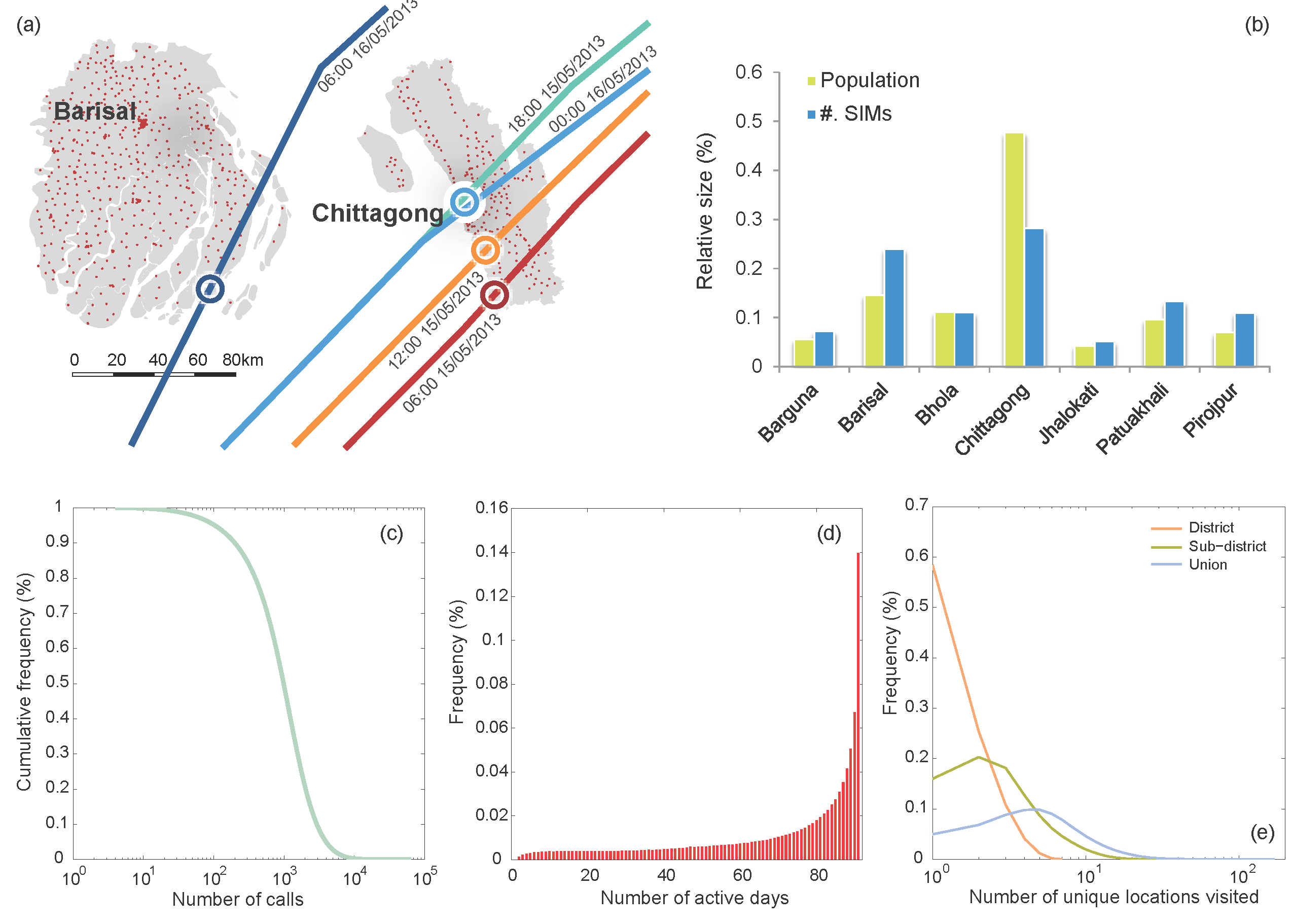


***Fig. S1*** *Study area, distribution of users, distribution of calls, number of active days, and unique locations visited. a) Map of study area, including tower map and cyclone path. Colored lines represent the forecasted versus actual cyclone paths. Forecasts predicted landfall over Chittagong, but on its final approach, Mahasen passed over Barisal. Actual versus predicted storm paths allow us to distinguish anticipatory and actual behaviors. b) Distribution of population and number of SIM cards at district level. Population data are taken from the 2011 census, and SIM cards’ locations are determined by the district in which they appeared most frequently during evening (10:00pm-6:00am) during the study period. c) A distribution of the number of calls per user shows that 90% of users made at least 200 calls during the three months period, and 50% of users made more than 1000 calls during the period. d) The number of active days on the network shows that 89% of users were active on 30 days, and 50% of users were active on at least 80 days. e) A distribution of unique locations visited shows that 95% of users visited more than one union.*

A comparison of number of SIM cards to population shows high agreement between the number of subscribers and the census figures (Fig. S1b). Deviations are seen in Chittagong district. In Bangladesh, household possession of a mobile phone grew from 78% of all households in 2011 to 89% in 2014, exceeding usage in other countries of similar socioeconomic profiles (National Institute of Population Research and Training (NIPORT) 2015). Users from the Grameenphone network, the largest in Bangladesh, numbered at 42 million in 2013, and comprised 61% of all mobile users nationwide (Telenor 2013). In this dataset, 90% of users made more than 200 calls during the study period and were active 80 days or more out of the 90 day study period (Fig. S1c, d). Likewise, SIM mobility is high: 95% of users appeared in more than one union, 84% in more than one sub-district, and 42% in more than one district (Fig. S1e). For the purposes of this study, we assumed that behaviors exhibited in CDRs are representative for the entire population. While earlier research support this notion, more studies are needed to adequately understand the representativeness of mobile phone users in Bangladesh.

S2. Quantifying impacts and infrastructure resilience

The functioning of mobile network towers potentially provides an additional proxy for cyclone damage. We undertook an analysis of network function during the study period, investigating towers that were inactive during Mahasen. Because of the potential for damaged equipment and interruption of services, mobile operators invest heavily in network resilience. Therefore in the most powerful cyclones, disabled towers could indicate where damages are concentrated, and could provide a proxy for other infrastructural damages sustained.

In the analysis of towers with no calling activity, we see the same four events that appear in other sections. Focusing on Mahasen, we evaluated the number of inactive towers in Mahasen’s impact zone, i.e., towers that registered zero calls, calculated on an hourly basis (Fig. S2). The Grameenphone network held up very well. During Cyclone Mahasen, only 60 towers went offline during landfall followed by 120 towers going offline during the course of the following day. It is likely that rather than suffering damages, towers went offline the following day as power sources failed and reserve batteries became depleted. Notably, patterns of “offline” towers between Mahasen and a heavy downpour that occurred between 30 May and 2 June were remarkably similar (Fig. S2).

In contrast to anomalies in calling frequency, recharges and mobility, tower function does not represent behavioral information, but may provide a potential indicator on the distribution of environmental impact to infrastructure. Mahasen was a relatively weak storm, and as such registered only minor disruption, however during major events, data showing inactive towers or loss of tower function may also indicate the locations where damages are concentrated, helping focus impact assessment on the most damaged sites. Further research is required to make meaningful inferences about the relationship between network function and infrastructural damage.


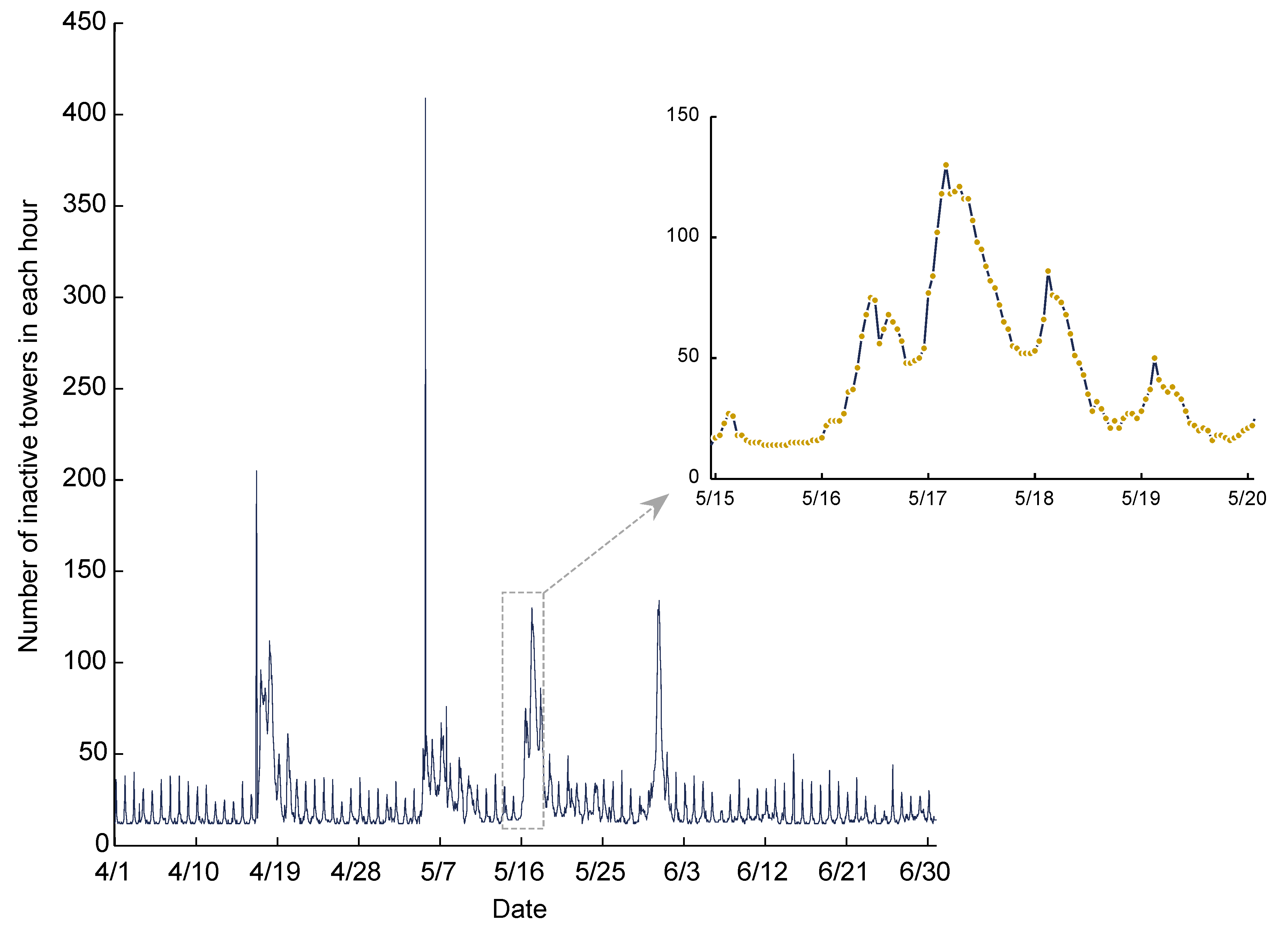


***Fig. S2*** *Number of towers with zero calls over the study period. For all filtered SIM cards, the number of calls received by each tower in each hour is calculated. Towers are classified as inactive during any particular hour if they received zero calls. There are four major periods when the number of inactive towers increased: 1) the Bengali New Year, 2) a national demonstration, 3) Cyclone Mahasen, and 4) a severe rainstorm.*

**References:**

National Institute of Population Research and Training (NIPORT), Mithra and Associates, and ICF_International (2015) Bangladesh Demographic and Health Survey 2014: Key Indicators. NIPORT: Dhaka, Bangladesh and Rockville, Maryland.

Telenor, (2013) Grameenphone, Bangladesh: In Bangladesh, Grameenphone is the leading provider of mobile communications services. <http://www.telenor.com/investors/company-facts/business-description/grameenphone-bangladesh/> Accessed 9 June 2015.
